# Protocol for evaluating the effects of a foot-ankle therapeutic exercise program on daily activity, foot-ankle functionality, and biomechanics in people with diabetic polyneuropathy: a randomized controlled trial

**DOI:** 10.1186/s12891-018-2323-0

**Published:** 2018-11-14

**Authors:** Renan L. Monteiro, Cristina D. Sartor, Jane S. S. P. Ferreira, Milla G. B. Dantas, Sicco A. Bus, Isabel C. N. Sacco

**Affiliations:** 1 Department of Physical Therapy, Speech, and Occupational Therapy, School of Medicine, University of São Paulo, São Paulo, Brazil; 2 Department of Physical Therapy, Federal University of Amapá, Amapá, Brazil; 3 Ibirapuera University, São Paulo, São Paulo Brazil; 4 University of Pernambuco, Petrolina, Pernambuco Brazil; 5 Department of Rehabilitation, Amsterdam UMC, Amsterdam Movement Sciences, Amsterdam, The Netherlands

**Keywords:** Diabetic neuropathies, Exercise, Diabetic foot, Foot ulcer, Clinical trial, Physical therapy

## Abstract

**Background:**

Diabetic polyneuropathy (DPN) negatively affects foot and ankle function (strength and flexibility), which itself affects the daily physical activity and quality of life of patients. A physical therapy protocol aiming to strengthen the intrinsic and extrinsic foot muscles and increase flexibility may be a promising approach to improve lower-extremity function, prevent further complications, and improve autonomy for daily living activities in these patients. Thus, the inclusion of a specific foot-related exercises focused on the main musculoskeletal impairments may have additional effects to the conventional interventions in the diabetic foot.

**Methods/Design:**

A prospective, parallel-group, outcome-assessor blinded, randomized controlled trial (RCT) will be conducted in 77 patients with DPN who will be randomly allocated to usual care (control arm) or usual care with supervised foot-ankle exercises aiming to increase strengh and flexibility twice a week for 12 weeks and remotely supervised foot-ankle exercises for a year through a web software. Patients will be evaluated 5 times in a 1 year period regarding daily physical activity level, self-selected and fast gait speeds (primary outcomes), foot ulcer incidence, ulcer risk classification, neuropathy testing, passive ankle range of motion, quality of life, foot health and functionality, foot muscle strength, plantar pressure, and foot-ankle kinematics and kinetics during gait.

**Discussion:**

This study aims to assess the effect of a foot-ankle strength and flexibility program on a wide range of musculoskeletal, activity-related, biomechanical, and clinical outcomes in DPN patients. We intend to demonstrate evidence that the year-long training program is effective in increasing gait speed and daily physical activity level and in improving quality of life; foot strength, functionality, and mobility; and biomechanics while walking. The results will be published as soon as they are available.

**Trial registration:**

This study has been registered at ClinicalTrials.gov as NCT02790931 (June 6, 2016) under the name “Effects of foot muscle strengthening in daily activity in diabetic neuropathic patients”.

**Electronic supplementary material:**

The online version of this article (10.1186/s12891-018-2323-0) contains supplementary material, which is available to authorized users.

## Background

Foot disorders are a major issue related to diabetic polyneuropathy (DPN) [1, 2]. Several sensorial and motor dysfunctions are directly related to ulcer formation and amputation [2]. Recent papers that focused on musculoskeletal foot-ankle complications and strategies to overcome them have been inconclusive [3–7] in defining the best strategy in preventing chronic complications related to DPN.

The progression of DPN affects the integrity of small joints and intrinsic muscles [3, 8–12]. These effects are the main factors for the development of deformities, elevated plantar pressures, and increased risk of plantar ulceration [8, 13–17]. These alterations affect the dynamic stability of the foot, generating an inadequate mobility for daily living activities [11, 12, 18, 19].

Recent guidelines for treating and preventing diabetic foot complications are based on the management/control of diabetes, integrated foot care, patient education, and self-management of foot care [20]. Besides these, foot orthosis and special shoes are recommended for reducing tissue mechanical stress and injuries [20]. Considering other rehabilitation approaches, including exercise therapy, showed to be beneficial in diabetic foot outcomes, particularly in increasing nerve velocity conduction of the lower limbs. Additional benefits can be induced by exercise in diabetic patients, such as skin sensitivity and intraepidermal nerve fiber density, which can delay the usual course of DPN, delay skin damage and ulceration [21]. Specific foot-ankle therapeutic exercises, have also shown promising results for improving sensitivity, foot-ankle range of motion and DPN symptoms [22], as well as for redistributing plantar pressure during locomotion [21, 23], but these are not part of the guidelines and require adequate investigation in well-designed studies prior to complementary recommendation in integrated care [20]. Many foot and lower limb disorders that result from diabetes, such as deformity, muscle weakness, decreased range of motion, rigidity of connective tissue, poor balance, and coordination, can potentially be restored or prevented by specific interventions. These neuromusculoskeletal alterations are common in DPN patients, and interventions such as strengthening, stretching, balancing, and gait training may be beneficial in preventing foot ulcers and amputation, fall risk reduction, improvement of daily physical activity level and quality of life, which can all reduce mortality and comorbidity rates.

Previous studies have reported the benefits of foot-ankle therapeutic exercises. A protocol performed at home for 1 month reduced peak plantar pressures during gait in DPN patients [24]. Likewise, personalized foot-ankle therapeutic exercise protocols to strengthen foot-ankle muscles showed positive results in satisfactorily redistributing plantar pressures during gait [4, 24], resulting in a better physiological pattern in foot-ankle rollover, and improvement of clinical measures of balance control [21].

DPN is also strongly associated with an inability to perform physical daily living activities, altered gait biomechanics, and increased number of falls [25]. Previous studies discussed diabetic patients’ reduced activity levels [26–29], which are important not only for glycemic control and cardiovascular health, but also patient mobility, as persons with diabetes are twice as likely to have mobility limitations compared to non-diabetics [30]. Tuttle et al. [28] showed that the number of steps of DPN patients are inversely proportional to the amount of intramuscular adipose tissue, suggesting that muscular impairment is caused by decreased physical activity levels. Motor and sensory deficits [31] and impaired foot range of motion [32, 33] severely reduce gait speed, affecting quality of life [31]. Unfortunately, there has not yet been any study reporting on the clinical effects of a specific foot-ankle exercise program and on falls incidence and daily physical activity levels.

Although there is evidence of profound changes in foot structure and function in DPN patients, whole body strengthening programs mostly neglect distal muscle groups, such as the ankle extrinsic and foot intrinsic muscles. The primary objective of this randomized controlled trial (RCT) is to investigate the effects of a 12-week therapeutic foot and ankle exercise program on daily physical activity level and self-selected and fast gait speeds at 12 weeks and after 1 year follow-up in patients with DPN. The secondary objectives of this study are to investigate the effects of this intervention at 6, 12, and 24 weeks and 1 year on foot ulcer incidence, ulcer risk classification, sensitivity, DPN symptoms, quality of life, foot health and functionality, foot muscle strength, and gait biomechanics.

## Hypotheses

Our hypotheses are that a 12-week foot-ankle therapeutic exercise protocol will:

- H 1. Increase daily physical activity levels
- H 2. Increase self-selected and fast gait speeds
- H 3. Reduce foot ulcer incidence in 1 year
- H 4. Not increase ulcer risk classification
- H 5. Increase foot tactile sensitivity
- H 6. Increase foot vibration sensitivity
- H 7. Decrease tactile sensory threshold
- H 8. Increase passive ankle range of motion
- H 9. Reduce DPN symptoms
- H 10. Improve health-related quality of life
- H 11. Improve foot health and functionality status
- H 12. Increase foot muscle strength
- H 13. Improve plantar pressure distribution
- H 14. Produce beneficial biomechanical changes during gait that denote an improvement in the mechanical efficiency of absorbing loads and propelling the body while walking and improve foot-ankle mobility. Such changes would include an increase in [1] the foot-ankle range of motion during stance phase, [2] ankle extensor moment and concentric power during propulsion phase, and [3] ankle flexor moment and eccentric power during heel-strike phase.

## Methods/Design

### Overview of the research design

This study is designed as a two-arm parallel-group, outcome-assessor blinded RCT that is prospectively registered in Clinical Trials number NCT02790931. The trial follows all recommendations established by SPIRIT [34].

The trial will be conducted in patients with DPN who are randomly allocated to:

- Control group (CG) - patients will not receive any specific intervention beyond usual care, which includes treatment recommended by the medical team, pharmacological treatment, and self-care guidelines, which are maintained in both groups [20].
- Intervention group (IG) - patients will receive usual care with additional foot-ankle exercises supervised by a physiotherapist twice a week and remotely-supervised exercises through Educational Diabetic Foot Software (SOPeD) twice a week for 12 weeks. After the 12-week period, the IG will continue exercising for the completion of the study (9 months) using the remotely supervised web software twice a week. http://www.usp.br/labimph/soped/

Patients of both groups will be evaluated five times in a 1 year period: at baseline (T0), after 6 (T6), 12 (T12), and 24 weeks (T24), and after 1 year (1y follow-up). All outcomes will be evaluated at each visit except for the biomechanical variables evaluated at T0 and T12. The primary outcome of daily physical activity will be evaluated at all instances except T6 for technical purposes.

**Fig. 1:**
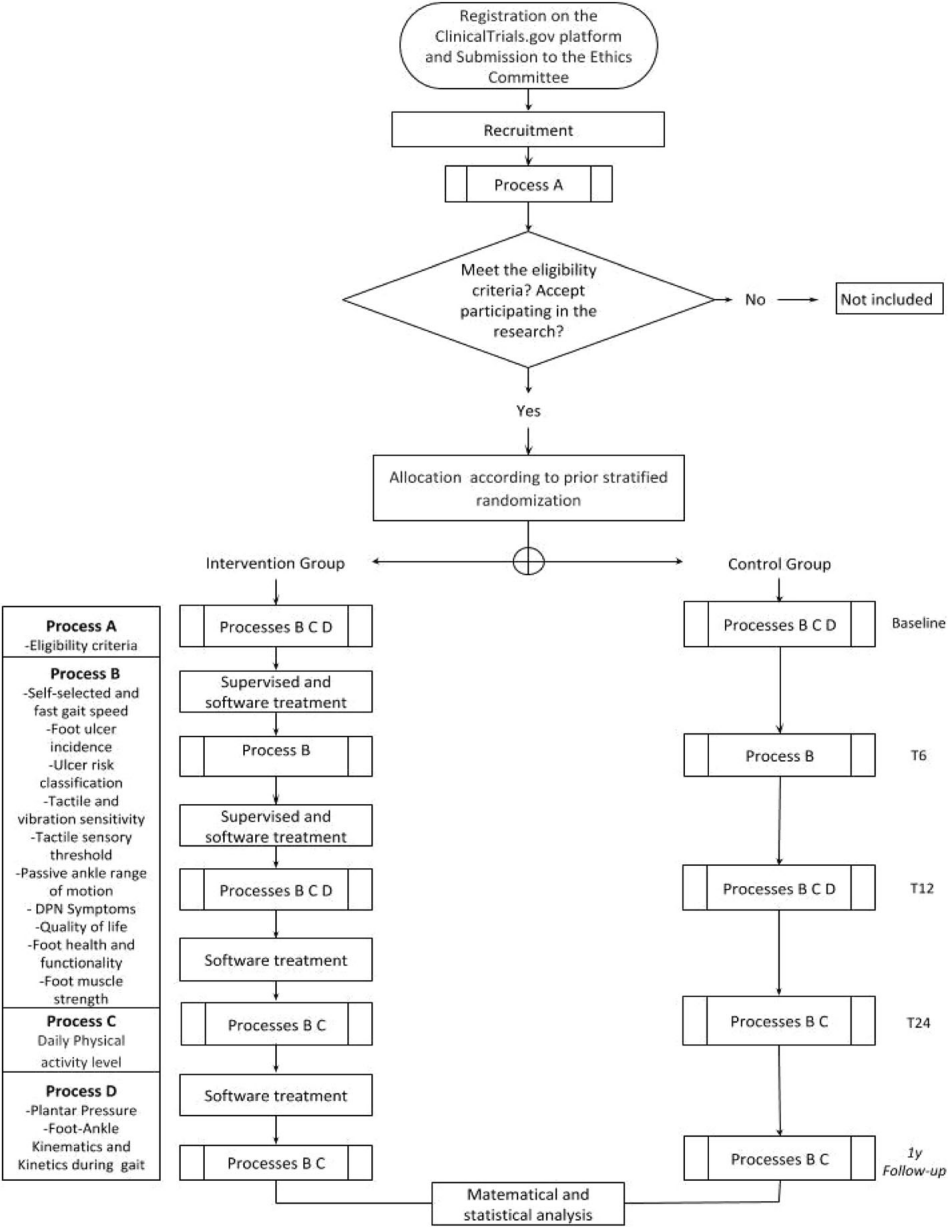
Flow chart illustrating the process of the study

The design and flowchart of the protocol are presented in Fig. 1. All procedures of this study follow the norms of an Operational Procedure Manual developed specifically for this research. The study will be conducted at the outpatient physiotherapy clinic of the primary care center *Centro de Saúde Escola Barra Funda Dr. Alexandre Vranjac* and the assessments will be performed at the *Laboratório de Biomecânica, movimento e postura humana* (LaBiMPH) at the Physical Therapy, Speech and Occupational Therapy department of the School of Medicine of the University of São Paulo, São Paulo, Brazil.

### Participants and recruitment

This study is currently recruiting patients (study start date: December 2017).

The inclusion criteria are:

- Either gender
- Adults up to 75 years
- Diabetes Mellitus type 1 or 2 diagnosed, with moderate or severe DPN confirmed by a Fuzzy software [9]
- Independent walking ability for at least 10 m
- A maximum of one amputated toe, not being the hallux
- Accessibility to electronic devices with internet that allow access to the web software

The exclusion criteria are:

- Presence of an active plantar ulcer
- History of surgical procedure at the knee, ankle, or hip or indication of surgery throughout the intervention period
- Arthroplasty and/or orthosis of lower limbs or indication of lower limb arthroplasty throughout the intervention period
- Diagnosis of neurological diseases
- Dementia or inability to give consistent information
- Receiving any physiotherapy during the intervention period
- Major vascular complications and/or severe retinopathy

### Randomization, allocation, and blinding

Both groups will be stratified according to the degree of DPN and gait speed, since both variables can highly influence clinical and biomechanical outcomes. Stratification will be performed according to the degree of DPN indicated by Fuzzy software (2–7.5: moderate degree of DPN, 7.6–10: severe degree of DPN) [9] and gait speed (slow: < 1.1 m/s, fast: ≥ 1.1 m/s) [35].

The randomization schedule will be prepared using Clinstat software (University of York, UK) by an independent researcher (Researcher #1) who will not be aware of the numeric code for the CG and IG groups. This sequence will be generated in blocks of random sizes [1–8] with random orders. The numerical sequence will be kept in opaque envelopes, numbered sequentially, following an order generated by the software. The randomization procedure will follow the instructions of Randelli et al. [36]. This sequence will be kept private and stored in a location where blind assessors do not have access.

After receiving the patients’ informed consent to participate, the random allocation to either the intervention or control group will be made by another independent researcher (Researcher #2), who will also be unaware of the codes. Only the physiotherapist (Researcher #3), responsible for locally supervised training, will know the group allocation of participants. Researcher 3 will also be responsible for the remote monitoring of the web software training. All patients’ personal data will be kept confidential before, during, and after the study by encoding participant’s names. Only the physiotherapist and the person receiving treatment will be aware of the meaning of each code. Patients will be allocated to study groups 1 week after baseline evaluation. The envelope with the initially-generated numerical sequence will then be opened, signed, and dated by the independent researcher, who will make the allocation (Researcher 2). Four physiotherapists (Researchers #4), also blind to treatment allocation, will be responsible for all clinical, functional, and biomechanical outcome assessments.

To guarantee the blindness of Researcher 4, before each evaluation, patients will be instructed not to reveal whether they are in the CG or IG; their questions should be asked only of the physiotherapist who is treating them (Researcher #3). The data tabulation and processing and trial statistician will also be blind to treatment allocation until completion of the main treatment analysis.

### Treatment arms

CG patients will not receive any specific intervention beyond treatment recommended by the health care team, which includes pharmacological treatment and self-care guidelines, and which will be maintained in both groups. The self-care guidelines adjusted for our setting in Sao Paulo include: performing daily inspection of the feet, using socks without elastic and sewing, cutting the nails in a square shape, avoiding cutting calluses or blisters without supervision, avoiding walking barefoot or wearing shoes without socks or wearing slippers, and seeking medical assistance whenever identifying problems in their feet. The IG patients will receive a therapeutic foot-ankle exercise protocol for strengthening and improving functionality under the supervision of a physiotherapist twice a week for 12 weeks, and a series of foot-ankle exercises will be performed under remote supervision through SOPeD twice a week for the entire 1-year study period. The web software will include written descriptions, photos, and audiovisual resources for each exercise. The supervised therapeutic routine will take approximately 50 min to complete, and the remotely-supervised routine will take a maximum of 20 min at home.

### Intervention

#### Control group

Patients allocated to the control group will not receive any specific intervention other than the treatment recommended by the medical staff and consensus of the International Working Group on the Diabetic Foot (IWGDF) [20], which includes:

1. Examine the feet annually for signs or symptoms of peripheral neuropathy and peripheral artery disease.
2. Screen for a history of foot ulceration or lower-extremity amputation, peripheral artery disease, foot deformity, pre-ulcerative signs on the foot, poor foot hygiene, and ill-fitting or inadequate footwear.
3. Instruct patient to inspect feet and the insides of shoes daily, wash feet daily (with careful drying, particularly between the toes), avoid using chemical agents or plasters to remove calluses or corns, use emollients to lubricate dry skin, and cut toe nails straight across.
4. Provide education aimed at improving foot care knowledge and behavior, as well as encouraging the patient to adhere to this foot care advice.

#### Intervention group

Patients allocated to the IG will receive a foot-ankle therapeutic exercise protocol for muscle strengthening and improving functionality. Part of the exercise protocol will be performed twice a week under the supervision of a physiotherapist for 12 weeks. A series of foot-ankle exercises will also be performed twice a week, remotely supervised through SOPeD. After 12 weeks of supervised and remote intervention, patients will continue home exercise practice using SOPED twice a week until the end of the study (for an additional 9 months).

The simplicity and practicality of this exercise protocol is an excellent tool for the management of the diabetes musculoskeletal complications in the primary and secondary care of public health systems. Both protocols (SOPeD and supervised therapeutic exercises) were designed following the same criteria: (a) warming exercises, (b) strengthening of the intrinsic foot muscles, (c) strengthening of the extrinsic ankle muscles, and (d) functional exercises, such as balance and gait training.

The following muscle groups were targeted in both protocols:

- Medial-plantar aspect: abductor hallucis, flexor hallucis brevis, and adductor hallucis
- Lateral-plantar aspect: abductor digiti minimi, flexor digiti minimi brevis, and opponens digiti minimi
- Middle-plantar aspect: flexor digitorum brevis, quadratus plantae, lumbrical muscles, plantar interosseous, and dorsal interosseous muscles
- Dorsal-foot aspect: extensor digitorum brevis and extensor hallucis brevis

The following joints were targeted in both protocols:

- Interphalangeal, metatarsophalangeal, and ankle joints

Supervised treatment will include 8 to 15 exercises to guarantee the four previously described criteria throughout the duration of the protocol (Additional file 1: Table S1 - Protocol for evaluating the effects of a foot-ankle therapeutic exercise). To promote long-term participation, each supervised session will be conducted in groups of 5–8 participants [37], and the duration of a session will be at least 50 min.

Remote exercise protocols will have a total of 8 exercises combined to provide the four previously described criteria through the duration of the protocol. To avoid monotony and enhance motivation, the exercises will change from session to session, and the maximum duration of a session will not be more than 20 min. A number of studies with diabetic patients have been conducted using e-health technologies that allowed people to engage in activities in their preferred environment, thereby taking up less of the health professional’s time and decreasing demands on health centers [38].

The web software exercise protocol was developed to provide autonomy and reduce the need for professional supervision. It contains clear video instructions (as well as text and audio) and preserves the safety of the target population during exercise. Furthermore, it establishes training volume, progression criteria, and guidelines for discontinuing the protocol. This tool personalizes the progress of a foot-ankle exercise program based on individual capabilities, similar to conventional physiotherapy, through a visual analogue scale, represented by a ruler and faces, which quantifies the level of effort required to perform each exercise so that daily progress can be customized. If the effort score ranges from 0.0–2.0 on the visual scale, the patient progresses to the next level the following day; from 2.1–7.0, the patient advances to the next level after 2 days; and from 7.1–10, the patient returns to the previous level.

To make the software more motivational, it has many game components [39]. Thus, users are rewarded in various ways: after finishing each stage, completing the self-assessment, and performing all the exercises that week. Users are also rewarded for dedication and persistence, not just physical ability. Each exercise and its training volume will be progressively modified based on the patients’ needs.

According to Huijgen et al. [40], rehabilitation systems with remote supervision have good acceptance and similar adherence to supervised interventions, with about 13% loss in their remote intervention group versus 15% in the control group. The increased adherence to treatment at home and its effectiveness are likely due to the remote intervention enhancing patient motivation in addition to prescribing progressive exercises aligned with their needs.

Data on exercise practice and foot evaluation will be summarized by the software and made visible to the patient. In addition, patients’ responses to the exercise software will be stored and accessible to researchers at any time. If any subject fails to login to the web software for more than 5 consecutive days, an e-mail will automatically be sent asking the subject to login and report training data (or lack thereof) for the past week.

The discontinuation criteria for exercise during any session include cramps, moderate to intense pain, fatigue, dizziness, fear, or any other condition that exposes the patient to any discomfort. Subjects in both groups will be advised to avoid other concomitant types of care such as physical therapy, acupuncture, or unconventional medical treatment during the study. In cases where treatment is indispensable, the patient must advise the investigators.

### Assessments

**Fig. 2:**
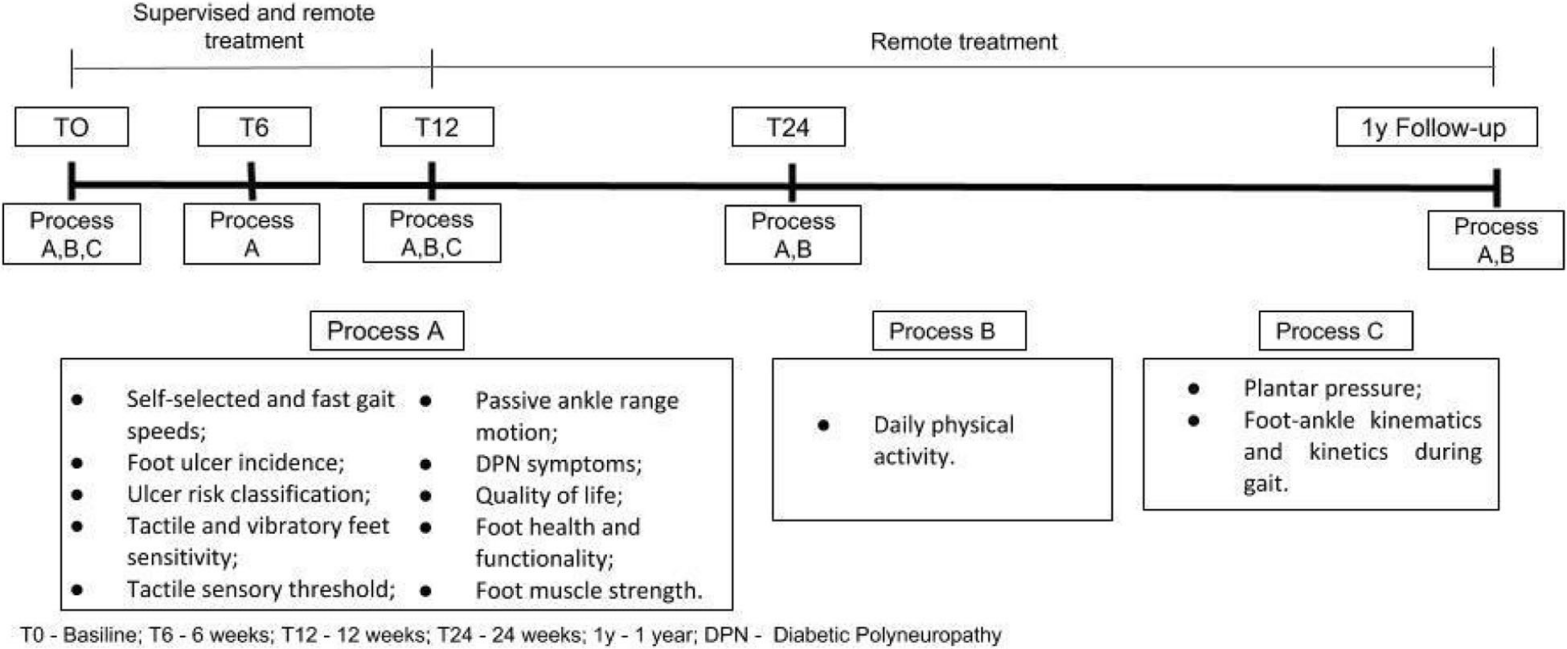
Timeline of the evaluation processes throughout the clinical trial

The scheme of evaluation processes is illustrated in Fig. 2. Four physiotherapists (Researchers #4) who are blind to group allocation will perform all assessments. The first assessment will consist of collecting personal details, anthropometry data, and all outcomes. After baseline assessment, all subjects will be scheduled for 4 assessments: at 6, 12, and 24 weeks, and at 1 year.

#### Primary outcomes

##### Daily physical activity level (number of steps)

Daily physical activity levels will be measured for 6 days by counting the number of steps using a 3D accelerometer (Power Walker-610, Yamax, Japan). This equipment measures the total number of steps and distance covered and has been previously validated with older people and patients with DPN [41, 42]. Before receiving the equipment, the accelerometer will be adjusted to the body weight and step length of each subject. To measure step length, the subject will be asked to walk comfortably in a 10-step interval. Thereafter, the mean step size will be calculated by measuring the 10 steps (toe to toe) divided by the number of steps. Each patient will be instructed to use the accelerometer daily, except during bathing and rest, for 6 days.

##### Self-selected and fast gait speeds

Patients will first walk barefoot on a 10 m track at a comfortable pace to determine self-selected gait speed and then as fast as possible to determine fast gait speed. For both speeds, 3 trials will be conducted, and the average will be calculated and used for analysis. Two photocells (CEFISE, Speed Test Fit Model, Nova Odessa, Brazil) located in the middle 6 m of a 10 m walking track will be used to measure walking time and calculate gait speed.

#### Secondary outcomes

##### Foot ulcer incidence

Throughout the study period, the presence of and moment of occurrence of plantar foot ulcers will be assessed. At each study visit (T0, T6, T12, T24, and Follow-up 1 year), two blind assessors will examine the entire surface of patients’ feet, including interdigital areas, to identify unreported or hidden foot injuries, in addition to asking the patient about foot wounds in the previous months since the last study visit. If an ulcer occur either during the intervention or the follow-up period, two blind assessors will check photographs of the patient’s foot and define if the occurrence is indeed an ulcer. Therapists will teach patients to inspect their own feet every morning to identify any evidence of skin lesions (e.g., abrasions, lacerations, blisters, and macerations) at or below the malleolus. A diabetic foot ulcer is defined as a “full thickness lesion of the skin distal to the malleoli in a person with diabetes mellitus” [43].Patients will be instructed to contact the research team immediately if these lesions are identified [29]. If a patient develops a plantar foot ulcer during the study, the intervention will be discontinued, and the patient will be followed up with the intention-to-treat analysis.

##### Ulcer risk classification

Ulcer risk classification will be performed according to the International Working Group on the Diabetic Foot (IWGDF) [43], in which Group 0 (no risk) patients do not present with DPN, with or without deformity, Group 1 (low risk) patients with only DPN, Group 2 (high risk) DPN patients with foot deformity or vascular disease, and Group 3 (severe risk) DPN patients with a history of foot ulceration or amputation. The presence of DPN will be assessed by Fuzzy software developed by our group and published previously [9], and the peripheral arterial disease will be classified using the ankle-brachial index defined by Boulton et al. [44]. Values less than 0.5 indicate severe vascular disease, 0.5–0.9 indicate vascular disease, and 0.9–1.2 are considered normal.

##### Tactile sensitivity

Tactile sensory deficits will be assessed by a 10 g monofilament [44, 45] in four plantar areas (plantar surface of the hallux and heads of the 1st, 3rd, and 5th metatarsals). This instrument has good reliability and validity in elderly individuals [46]. The monofilament will be applied perpendicularly to the skin surface 3 times on the tested areas with sufficient force to cause the filament to bend or buckle. The sequence of the tested areas will be randomized. The patient will not be able to see the monofilament or where it is being applied. The number of areas in which the patient does not feel pressure will be recorded [47]. The greater the number of areas marked, the greater the impairment of tactile sensitivity.

##### Vibration sensitivity

Vibration testing will be conducted with the timed method using a 128 Hz tuning fork applied to the dorsal surface of the distal phalanx of the hallux. The time (in seconds) at which vibration sensation diminishes beyond the examiner’s perception will then be recorded from both sides on a standardized form [48]. Values ​​less than 10 s are classified as present vibratory sensitivity, ​​greater than 10 s are classified as decreased vibratory sensitivity, and no perception is classified as absent vibratory sensitivity.

##### Tactile sensory threshold

The tactile sensory threshold will be assessed in the dorsal surface of the hallux using 6 monofilaments: 0.05 g, 0.2 g, 2 g, 4 g, 10 g, and 300 g. Patients will lay in prone position with the leg resting comfortably on a stretcher. Both feet are evaluated. Monofilaments are applied in order of increasing stiffness. A positive threshold will be recorded when the subject can feel the filament [49].

##### Passive ankle range of motion

The passive ankle range of motion will be evaluated bilaterally using an ankle electrogoniometer (model SG110/A, Biometrics, Gwent, UK). The biaxial electrogoniometer has two endblocks: a mobile (telescopic) and a fixed block joined by an instrumented spring with strain gauge. These endblocks attach to the ankle joint. The fixed endblock is positioned parallel to the major axis of the foot, below the lateral malleolus, and the telescopic endblock is aligned with the major axis of the leg. The strain gauge spring is kept tense and its center is coincident to the ankle joint axis (over the lateral malleolus) with the sensor attached to the subject. The system is calibrated with the ankle in its mechanical neutral position: standing in a relaxed posture in stationary equilibrium, with the body weight distributed equally between the feet and the output value defined as the zero angle of the goniometer. Forward motion of the lower segment is regarded as flexion (negative values) and backward motion as extension (positive values) [50]. After setting the zero angle, the patient will lie down and the assessor will measure the passive range of motion.

##### DPN symptoms

Patients will answer the Brazilian version of the Michigan Neuropathy Screening Instrument (MNSI) [51]. This questionnaire has 15 questions about the sensitivity of the legs and feet and is self-administered. Answers of “yes” for questions 1, 2, 3, 5, 6, 8, 9, 11, 12, 14, and 15 receive a score of 1. A “no” answer for questions 7 and 13 score 1. Question 4 is a measure of circulatory deficit and question 10 is a measure of general asthenia and are not included in the score. The sum of all scores ranges from 0 to 13 (13 representing a worse DPN).

##### Quality of life

Patients will answer the EQ-5D questionnaire [52], which is a generic instrument for measuring health-related quality of life that allows the assessor to generate an index representing the individual’s health status. It is based on a classification system that describes health in five dimensions: mobility, personal care, usual activities, pain/discomfort, and anxiety/depression. Each dimension has three associated severity levels, corresponding to no problems (level 1), some problems (level 2), and extreme problems (level 3). The EQ-5D associates a value between − 0.59 and 1.00, which represents the health status of a patient (1 being perfect health).

##### Foot health and functionality

This study will use a Brazilian-Portuguese version of a foot-health status questionnaire (FHSQ-BR) translated and validated by Ferreira et al. [53]. Section I evaluates foot health in four domains: foot pain, foot function, footwear, and general foot health. Section I is composed of questions with answer options presented in affirmative sentences and corresponding numbers. Section III collects general demographic data. This study will only use the scores from Section I because Section II refers to general health. Each domain scores from 0 to 100 points, where 100 is the best condition and 0 the worst. The Scores will be calculated using the FHSQ software version 1.03 (Care Quest, Australia).

##### Foot muscle strength

Foot muscle isometric strength will be measured according to Mickle et al. (2006) [54] using a pressure platform (emed q-100, Novel, Munich, Germany). Subjects will stand and push down on the platform two times, as hard as possible, with their hallux and toes, which controls for excessive body sway. The maximum force under the hallux and toes normalized by bodyweight are outcomes of this measurement.

##### Dynamic plantar pressure distribution during gait

A 700 × 403 × 15.5 mm pressure platform (emed q-100, Novel, Munich, Germany) with 6080 sensors and 4 sensors per cm^2^ that collects data at 100 Hz will be used to assess walking plantar pressure distribution. Participants will walk barefoot to the platform with a self-selected gait speed three times for 4 m. Both feet will be analyzed for each patient. Based on the algorithm by Giacomozzi et al. [55], peak pressure, contact area, and pressure-time integral in seven anatomical plantar regions will be analyzed: heel, midfoot, medial forefoot, medium forefoot, lateral forefoot, hallux, and toes. This method relies on the integration of a 3D motion capture system (Vicon system), a pressure measurement device (emed q-100), a multi-segment foot model, and an algorithm to identify regions of interest.

##### Foot-ankle kinematics and kinetics during gait

Gait kinematics will be acquired using three-dimensional displacements of passive reflective markers (9.5 mm in diameter) tracked by eight infrared cameras at 100 Hz (VERO, Vicon Motion System Ltd., Oxford Metrics, UK) and the NEXUS 2.6 motion capture software (Vicon Motion System Ltd., Oxford Metrics, UK). Three-dimensional and force-platform motion capture data will be collected to quantify the magnitude and direction of biomechanical responses during gait. Forty-three markers will be placed on the subject (leg, ankle, and foot) according to the Oxford protocol.

The laboratory coordinate system will be established at one corner of the force plate and all initial calculations will be based on it. Each lower-limb segment (shank and thigh) will be modeled based on surface markers as a rigid body with a local coordinate system that coincides with the anatomical axes. Translations and rotations of each segment will be reported relative to the neutral positions defined during the initial static standing trial. All joints will be considered to be spherical (i.e., with three rotational degrees of freedom). Ground reaction forces will be acquired by a force plate (AMTI OR-6-1000, Watertown, MA, USA) with a sampling frequency of 1 kHz embedded in the center of the walkway. Force and kinematic data acquisition will be synchronized and sampled by an A/D board (Control Box LOCK VICON, 192 kHz, 24 bits).

Five valid steps will be acquired from the same foot as the pressure distribution measurements on a 10 m walkway. The bottom-up inverse dynamics method will be used to calculate the ankle force moments in the sagittal plane, considering the inertial properties of segments [56]. For the calculation of ankle power, the calculated moment of force and the angular velocity of the ankle in the sagittal plane will be considered. Calculation of all variables will be performed using a custom-written MATLAB function (MathWorks, Natick, MA, USA).

The kinematic and kinetic outcomes that will be analyzed are: (a) the total sagittal plane ankle range of motion during gait stance phase (degrees); (b) the ankle angle in three planes at the heel strike (degrees); (c) the ankle angle in three planes at the final phase of push off (degrees); (d) range of dorsiflexion during gait stance phase (degrees); (e) dorsiflexor ankle moment peak at the heel strike and approximately 80% of gait support phase, corresponding to the beginning of the propulsion; (f) the ankle power peak at approximately 80% of the stance phase (W/kg) corresponding to the propulsion phase; (g) deformation of the medial longitudinal arch angle; (h) rotation between forefoot and rearfoot; (i) angle in the transversal plane between first and second metatarsals and between second and fifth metatarsals; and (j) maximum inversion and eversion (frontal plane).

##### Evaluation of the outcome-assessor blinding

To evaluate whether or not there was a failure in blinding of the outcome assessor, assessors will be asked to guess which group the patients belonged to at the end of 12 weeks of treatment. Then the evaluators will classify the certainty of their opinions according to a scale (1 = not sure, 5 = completely sure). To ensure that the evaluator is not induced to correctly guess the participants’ allocation, the patient will be instructed to not disclose any behavior details during the previous 12 weeks.

### Outcome measurements

The outcome measurements are described in Table 1.

**Table 1:** Outcomes Measurements

Outcome		When will they be evaluated				
		*Baseline*	6 weeks	12 weeks	24 weeks	*Follow-up* 1 year
Primary	Measures					
Daily Physical Activity level	Number of steps by Accelerometers	X		X	X	X
Self-selected and fast gait speed	Speed in m/s measured by Photoelectric Cells	X	X	X	X	X
Secondary	Measures					
Foot ulcer incidence	Number of new cases of ulcers in 12 months of the study	X	X	X	X	X
Ulcer risk classification	Classification according to IWGDF	X	X	X	X	X
Tactile sensitivity	Number of non-sensitive areas measured by 10 g monofilaments	X	X	X	X	X
Vibration sensitivity	Classification the ability to feel the vibration measured by tuning fork	X	X	X	X	X
Tactile sensory threshold	Tactile sensitivity threshold between different monofilament thicknesses	X	X	X	X	X
Passive ankle range of motion	Ankle angle measured by a digital electrogoniometer	X	X	X	X	X
DPN Symptoms	Score of Michigan Neuropathy Screening Instrument (MNSI)	X	X	X	X	X
Quality of life	Score of EQ-5D questionnaire	X	X	X	X	X
Foot health and functionality	Scores of FHSQ-BR questionnaire	X	X	X	X	X
Foot muscles strength	Maximum force obtained on EMED pressure platform	X	X	X	X	X
Plantar pressure	Peak pressure obtained on EMED pressure platform	X		X		
Foot-ankle kinematics and kinetics during gait	Three-dimensional motion capture and a force platform	X		X		

### Sample size and statistical analysis

The sample size was calculated using the GPower v. 3.1 program [57] based on the following outcomes: daily physical activity level (number of steps) and self-selected and fast gait speeds. These three outcomes were chosen because they reflect important functional gains for patients with DPN. Thus, three sample calculations were performed and selected, which resulted in the largest number of participants. For fast gait speed, effect size was calculated based on a study that evaluated the effect of exercise on the fast gait speed in elderly patients, which had an increase in gait velocity from 151.9 ± 5.5 to 162.7 ± 6.9 cm/s after 3 months of intervention [58]. For self-selected gait speed, the effect size was based on the minimal clinical difference in self-selected gait speed (0.17), as it may be useful for establishing therapeutic goals and interpreting patient progress to treatment [59]. For number of steps, effect size was calculated based on a study that evaluated the effect of interactive balance training on daily number of steps in individuals with DPN, for which there was an increase from 8.656 ± 4.589 to 11.052 ± 5.365 after 4 weeks of intervention [30].

Considering the primary outcome tested; a statistical design of F-test repeated measures and interaction between and within factors with 3 repeated measures and two study groups; a statistical power of 0.80; an alpha of 0.05; and a size of effect of 0.175, 0.170, and 0.154 for fast gait speed, self-selected gait speed, and number of steps, respectively, the resulting sample sizes were 54, 58, and 70 individuals, respectively. Therefore, the number of participants is based on the measurement for number of steps, which resulted in the largest number of participants (*n* = 70). Assuming a 10% dropout rate during the study, a sample size of 77 patients is needed.

Inferential statistical analysis will be done using an intention-to-treat and per protocol analysis. The missing data will be treated by imputation methods depending on the type: missing completely at random, missing at random, or not at random [60]. The per-protocol analysis will include only those patients who completed follow up in the allocated intervention group.

After confirmation of normality (Kolmogorov-Smirnov test), homoscedasticity (Levene test), and imputation of the means for the missing data of variables with normal distribution, ANOVA 2 factors for repeated measures will be performed, followed by Newman posttest Keuls, to obtain the group effect (intervention and control), time (between T0, T6, T12, T24, and Follow-up 1 year), and group x time interaction.

Significant differences will be considered with α = 5%, but for the description of the effect of the intervention, the effect size (Cohen coefficient) and difference between the means will be calculated with their respective 95% confidence intervals.

## Discussion

We have presented the rationale and design of a Randomized Controlled Trial on the efficacy of foot-ankle therapeutic exercise training in DPN patients. This RCT will provide important data on foot-ankle training effectiveness on daily physical activity levels and clinical and biomechanical outcomes. The outcomes may contribute to the design of future studies on clinical and biomechanical changes resulting from the strengthening of the foot-ankle complex.

Some studies have sought to evaluate the effects of strengthening on several outcomes in patients with DPN. Ten studies used generic lower limb exercises that did not focus specifically on musculoskeletal deficits related to diabetes: balance training, non-weight-bearing and weight-bearing strengthening, aerobic exercises, and multimodal manual treatment treatment [29, 30, 53, 61–67].Four studies that sought to evaluate the effects of specific foot-ankle training had methodological biases, such as lack of a control group [68], lack of DPN clinical outcomes [4], low number of participants [24], and short-term effects [25].

The RCT introduced here will have a longer period of follow-up (12 months), several clinical DPN outcomes, and a calculated sample size to achieve enough power within a cohort of patients with moderate and severe DPN. In addition, this trial proposes a specific training protocol for intrinsic and extrinsic foot-ankle muscle strengthening focusing on DPN deficits, including several easy-to-perform exercises that do not require continuous supervision by a health-care professional. The innovative and original exercise program presented in this RCT will be a promising approach to treat and prevent foot complications in this population and improve their autonomy for daily living activities.

## Additional file

Additional file 1: **Table S1.** Protocol for evaluating the effects of a foot-ankle therapeutic exercise. (DOCX 752 kb)

## Abbreviations

CG: Control group
DPN: Diabetic polyneuropathy
FHSQ-BR: Brazilian-Portuguese version of a foot-health status questionnaire
IG: Intervention group
IWGDF: The international working group on the diabetic foot
LaBiMPH: *Laboratório de Biomecânica, movimento e postura humana*
MNSI: Michigan neuropathy screening instrument
RCT: Randomized controlled trial
SOPeD: Educational diabetic foot software
T0: Baseline
T12: 12 weeks
T24: 24 weeks
T6: 6 weeks
