# Supplementary material for: Protocol for evaluating the effects of a foot-ankle therapeutic exercise program on daily activity, foot-ankle functionality, and biomechanics in people with diabetic polyneuropathy: a randomized controlled trial
Source: BMC Musculoskelet Disord. 2018 Nov 14;19:400. doi: 10.1186/s12891-018-2323-0 (PMC6236874; doi:10.1186/s12891-018-2323-0)
Supplement: Supplementary file 1 — Table S1. Protocol for evaluating the effects of a foot-ankle therapeutic exercise. (DOCX 752 kb) [file 12891_2018_2323_MOESM1_ESM.docx]

**Additional file 1**

**Short title: Protocol for evaluating the effects of a foot-ankle therapeutic exercise**

**Table S1:** Exercise protocol

**WARMING EXERCISES**

| **Exercise** | **Performance** | **Volume and progression** | **Approximate total duration** |
| --- | --- | --- | --- |
| 1. Stretching of the sole of the foot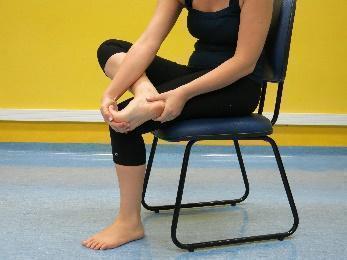 | Sitting, cross your leg over your knee. With one hand, pull your toes back. With the other hand, massage the area on the bottom of your foot just in front of your heel. | Massage 1 min  each foot | 2 – 3 min |
| 2. Massage with the ball  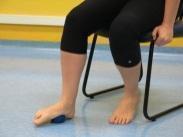 | Sitting, put a ball under your foot and massage back and forth (forward and backward); To one side and the other. | Massage 1 min  each foot. | 2 – 3 min |
| 3. Move your feet up, down and in circles  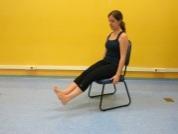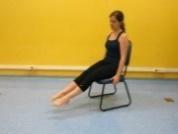  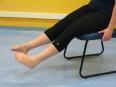 | Sitting, move your feet up and down, and then move in circles. First: flexion and extension exercise. Second: Clockwise circles Third: Counterclockwise Circles | 1: 1x10 rep;  2: 2x10 rep;  3: 1x10 rep;  4: 2x20 rep. | 3 – 4 min |
| 4. Writting words with your feet | Sitting down, write words in the air with your feet. | __ | __ |
| 5. Calf muscle stretching  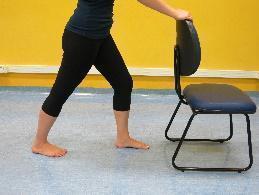 | Standing in front of a chair or wall, keep one leg in front of the other. The front leg with the knee flexed and the rear leg withe the knee extended. Lean forward at the ankle, keeping both heel on the ground, stretching the calf muscles. | 1 x 20 s each  leg. | 1 min |
| 6. Support on the lateral and medial border of the foot  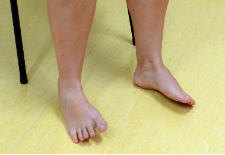 | Sitting, knees bent and feet flat on the floor. Support both feet by the lateral edge of the foot, followed by the support of the medial edge of the foot. | 1:1x10 rep holding each position for one second.  2:2x10 rep;  3:2x20 rep. | 2 – 6 min |
| 7. Interlacing your fingers and toes and making circular movements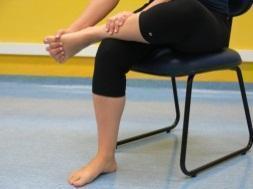 | Sitting, cross one leg over the other and interlacing your fingers on your toes and perform circular motions. | 1 x 20 s each foot. | 1 min |
| 8. Toes Manipulation  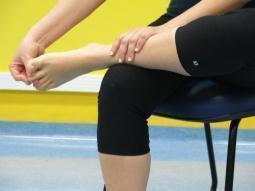 | Sitting, with one leg crossed over the other, hold each toe and slowly spin side to side, like a screw. Do it all your toes. | 1x 15 rep each toe | 1-2 min |
| 9. Massage with the ball without contact of the heel  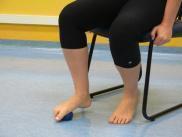 | Sitting, place a ball under your foot and press it toward the floor. Do not place the heel on the floor. | Press during 1 min  each foot. | 2-3 min |
| 10. Self-Massage in the feet 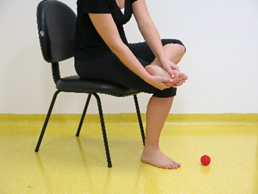 | Sitting with one leg crossed over the other, massage the soles of your feet with both hands for 20 seconds. In a circular motion using the thumb, go in the direction of the heel up to the fingers. Do the same with the other foot. | 1 x 20 s | 1 min |
| 11. Alternate toe support (sitting)  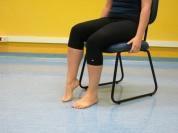 | Sitting on a chair, stand on tiptoe, alternating feet. | 1:1x10 rep  2:2x10 rep  3:2x20 rep | 1 – 4 min |

**INTRINSIC MUSCLES EXERCISES**

| **Exercise** | **Performance** | **Volume and progression** | **Approximate total duration** |
| --- | --- | --- | --- |
| 12. Toe alternate  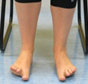 | Sitting, with the heel fixed and contacting the floor, alternately touch the first and fifth on the floor. Do not move your knees. Do it slowly and under complete control. | 1: 1x 10 rep;  2: 1x 10 (standing); | 2-3 min |
| 13. Pick up objects with your toes (1^st^ cotton / lapis / ball)  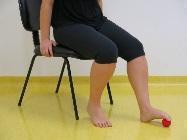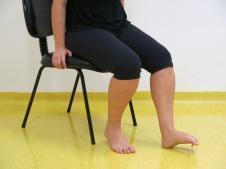 | After placing an object on the floor (cotton, ball and pencil), take it with your toes | 1:1x5 rep. holding for 5 seconds  2:2x5 (standing)  3: 3x5 (standing) | 2 – 3 min |
| 14. Wringing towel with feet  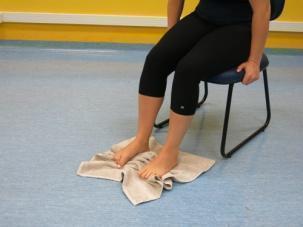 | Sitting, with the heel fixed and in contact with the floor, pull the towel with your toes without suspending the heel (Both feet) | 1: 1x10 rep;  2: 1x15 rep; | 1 – 2 min |
| 15. Open and close the toes (from the second to the fifth)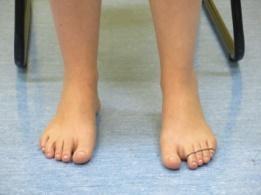 | With an elastic between the second and fifth toe, perform the opening / separating movement of the fingers against the resistance of an elastic | 1:1 x 10 rep (sitting).  2: 2x15 (standing)  3: 3x20 (Standing). | 1-2 min |
| 16. Squeeze toes separators  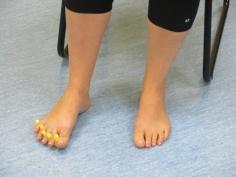 | Sitting, with 90 degrees of the knee and ankle flexion, adduct and abduct, squeeze the toes separators for one second Always keeping the heel fixed on the ground. | 1: 1x10 rep each  foot;  2: 2x10 rep;  3: 3x10 rep. | 1 min |
| 17. Toe toes  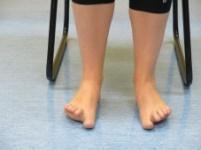 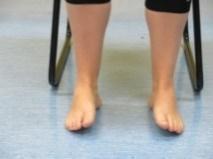 | Sitting on a chair with your feet flat on the floor, tap one toe at a time, starting with the little toe, on the floor continuously. Doing a similar movement while strumming. After performing the same movement starting with the big toe. | 1: 1x10 rep (sitting);  2: 1x10 (Standing);  3: 1x20 (Standing). | 2 – 6 min |
| 18. Toes flex with theraband  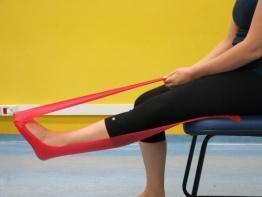 | Sitting, with the heel resting on the floor, flexion of the toes without moving the ankle. | 1: 1x10;  2: 2x10;  3: 2x20. | 1 min |
| 19. Plantar arch raise  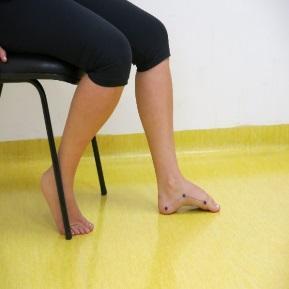 | Sitting, raise the plantar arch in na arch shape. The heel and fingertips should not get off the ground. | 1: 1x10;  2: 2x10;  3: 3x10. | 1-2 min |
| 20. Short-foot exercise | Sitting, with 90 degrees of knee and ankle. Approximate the head of the first metatarsal toward the heel without toe flexion,“ shortening“ the feet. The forefoot and heel should not get off the ground. | 1: 1x10;  2: 2x10;  3: 3x10. | 1-2 min |

**ANKLE EXERCISES**

| **Exercise** | **Performance** | **Volume and progression** | **Approximate total duration** |
| --- | --- | --- | --- |
| 21. Climb on the tip Feet  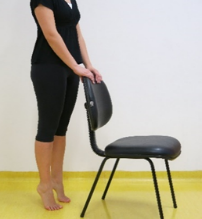 | Using a support, chair or any other stable furniture, stand on tiptoe and return to the starting position. | 1: 1x5 rep;  2: 1x10 rep;  3: 1x15 rep. | 1 – 2 min |
| 22. Kick the floor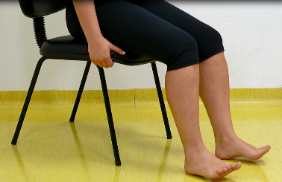 | Sitting with feet flat on the floor, tapping the front of the foot repeatedly on the floor, with a fast speed, as if "impatient." Do one foot at a time. | 1: 1x30 rep;  2: 2x30 rep;  3: 2x40 rep. | 2 – 3 min |
| 23. One Foot Balance  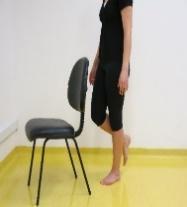 | Stand on one foot only. Do one side and then the other. | 1: 1x10 rep;  2: 2x10 rep;  3: 1x10 rep;  4: 2x20 rep. | 3 – 4 min |
| 24. Tighten the ball  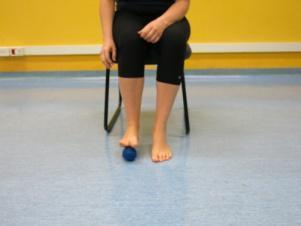 | Sitting, put your foot on a ball and press it down. The heel should rest flat on the floor. | 1: 1x10 rep;  2: 1x15 rep;  3: 1x20 rep. | 1 min |
| 25. Strengthening the medial musculature of the foot  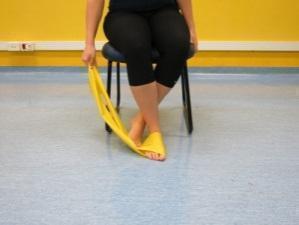 | Placing an elastic band around the medial part of the foot (below the big toe) and with the other foot stepping on the elastic band to give resistance. Make a movement against the elastic band, as if you would step on the floor with the lateral edge of the foot. | 1: 1x10 rep (yellow elastic band);  2: 1x10 rep (blue elastic band) | 1 – 2 min |
| 26. Strengthening the lateral musculature of the foot  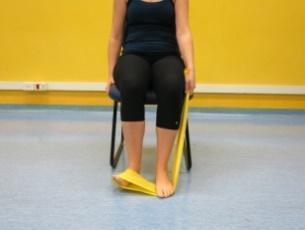 | Placing an elastic band around the lateral of the foot (below the little toe) and with the other foot stepping on the elastic band to give resistance. Make a movement against the elastic band, as it would walk with floor with the medial edge of the foot. | 1: 1x10 rep (yellow elastic band);  2: 1x10 rep (blue elastic band) | 1 – 2 min |

**FUNCTIONAL EXERCISES**

| **Exercise** | **Performance** | **Volume and progression** | **Approximate total duration** |
| --- | --- | --- | --- |
| 27. Walk with open toes  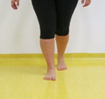 | Walk slowly keeping your toes apart as long as your foot stays flat on the floor. | 1: 1x10 rep;  2: 2x10 rep;  3: 2x20 rep. | 1 – 2 min |
| 28. Walking across the steps.  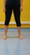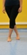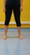 | Walk to the side by crossing one leg in front and then crossing back. Return side the same way to the starting position. | 1: 1x10 rep;  2: 2x15 rep;  3: 2x20 rep. | 2 – 3 min |
| 29. Step forward and backward  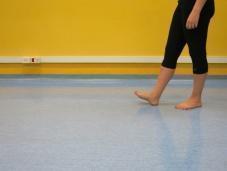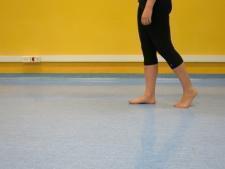 | Unload the weight forward and backward associated with ankle flexion and extension (Simulating the gait). | 1: 2x15 rep;  2: 2x20 rep;  3: 2x30 rep | 3 – 4 min |
| 30. Walk with your toes pressed to the floor  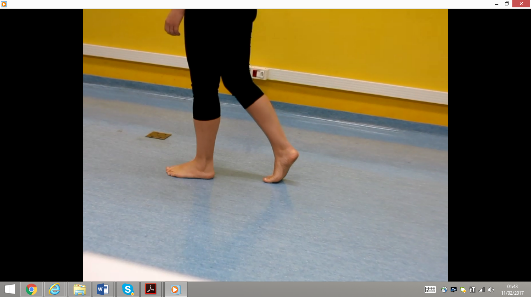 | Walk with your toes pressing toward the ground, as if you were pushing the ground with your toes as you walk. | 1: 1 x 10 steps, holding for 1 S  2: 2x10 steps;  3: 3x10 steps. | 2-3 min |
